# Novel human partner cell line for immortalisation of rare antigen-specific B cells in mAb development

**DOI:** 10.1186/1753-6561-5-S8-P130

**Published:** 2011-11-22

**Authors:** Galina Kaseko, Marjorie Liu, Qiong Li, Tohsak Mahaworasilpa

**Affiliations:** 1 The Stephen Sanig Research Institute, Suite G17, National Innovation Centre, Australian Technology Park, 4 Cornwallis Street, Eveleigh, NSW, 2015, Australia

## 

It is well-documented that post-translational modification (PTM) events, such as glycosylation, play an important role in antibody-dependent cell-mediated cytotoxicity (ADCC) [1,2]. In current technological processes, monoclonal antibody (mAb) production is widely achieved using heterologous hybridoma systems or genetic engineering using various non-human cell lines as expression host. As a consequence, PTMs generated from non-human cell lines may differ from their human counterparts, resulting in diminished antibody efficacy, aberrant folding and adverse immunogenic response. Therefore, the use of human partner cell lines to generate “fully human” mAbs is beneficial as it circumvents functional complications associated with non-human cell lines.

A human cross-lineage hybrid cell line was developed in our laboratory as a candidate partner for immortalisation of rare primary human antigen-specific B lymphocytes using binary electrical cell hybridisation technique which has been developed in house. This novel partner cell line is a tri-hybrid of IL-4 secreting Th2 lymphoblast derived from a patient with acute lymphoblastic leukaemia (T), CD20^+^ B lymphoblast, also derived from a patient with acute lymphoblastic leukaemia (W), and IL-6 secreting peripheral blood-derived CD14^+^ monocyte (M). The selection of cell phenotypes used to create the tri-hybrid was based on factors known to maintain and promote antibody production.

The resulting tri-hybrid (WTM) displayed characteristics of mixed CD phenotypes with the majority of cells being CD20^+^ (95%) with co-expression of CD4^+^ (54%) and CD14^+^ (24%). It secreted IL-4, IL-6, IL-8 and GM-CSF but was negative for IL-1A, IL-1B, IL-2, IL-5, IL-10, IL-12, IL-13 and IL-17. The cell line did not express the tumour suppressor protein, p53, and neither did it secrete immunoglobulins (Ig)/ Ig chains nor were they expressed on the surface.

WTM cells were then used as a fusion partner with primary antigen-experienced CD19^+^ B cells which had been isolated from peripheral blood, activated *in vitro*, and the resultant hybrids were sorted for IgM^+^ and IgG^+^ expression. 100% hybridisation success rate was achieved using a binary electrical cell hybridisation technique and the number of resulting stable hybrids varied from 48% to 78% depending on the phenotype of B lymphocytes used in experiments. 23% to 68% of those stable hybrids secreted Ig with production ranging between 0.2 to 1.2 ìg/10^6^ cells (Table 1). Cytokine screening of some of the Ig producing hybrids revealed a cytokine profile which was inherently different to that of the WTM partner cell line. The Ig producing hybrids concurrently expressed IL-10 and GM-CSF but not IL-4, IL-6 or IL-8. These hybrids were also positive for TGF-â, RANTES, MIP, MCP and MDC.

**Table 1:** Success rate of stable hybrid generation and Ig producing hybrids

Event	Success rate,	
Hybridisation	100	(%) of attempts
Stable hybrids	48	(%) of hybridised
Ig producing hybrids	23	(%) of stable hybrids

In conclusion, major advantages of our method involve the rapid generation of stable Ig producing hybrids from a small B lymphocyte population size (50 cells) and the elimination of conventional laborious screening methods for hybrids and Ig producing clones. Thus, when the number of rare antigen-specific B cells available is a limiting factor in generating hybridoma, EBV transfection or direct sequencing, binary electrical B lymphocyte hybridisation with WTM cells can provide a very attractive approach for the generation of stable hybrid cell lines producing monoclonal antibodies.
